# A cell-penetrating whole molecule antibody targeting intracellular HBx suppresses hepatitis B virus via TRIM21-dependent pathway: Erratum

**DOI:** 10.7150/thno.72666

**Published:** 2022-04-26

**Authors:** Jun-Fang Zhang, Hua-Long Xiong, Jia-Li Cao, Shao-Juan Wang, Xue-Ran Guo, Bi-Yun Lin, Ying Zhang, Jing-Hua Zhao, Ying-Bin Wang, Tian-Ying Zhang, Quan Yuan, Jun Zhang, Ning-Shao Xia

**Affiliations:** 1 State Key Laboratory of Molecular Vaccinology and Molecular Diagnostics, School of Public Health, Xiamen University, Xiamen 361102, China;; 2 National Institute of Diagnostics and Vaccine Development in Infectious Diseases, School of Life Science, Xiamen University, Xiamen 361102, China;; 3 School of Medicine, Shenzhen University, Shenzhen, 518060, China;; 4 Department of Clinical Pathology, Affiliated Hospital of Guangdong Medical College, Zhanjiang, 524001, China.

In the original publication, errors were found in Figure 2E. During the assembling of images in Figure 2E, the panel images of FLP and the merged image of CYTD were misused with the 9D11 images from Figure 2C; the images of the control panel were cropped incorrectly. The authors have checked all of the source data, the correct figures are shown below. The authors confirm that these corrections do not change the result interpretation or conclusions of the article. The authors are deeply sorry and sincerely apologize for any inconvenience or misunderstanding that may have been caused.

## Figures and Tables

**Figure 2:**
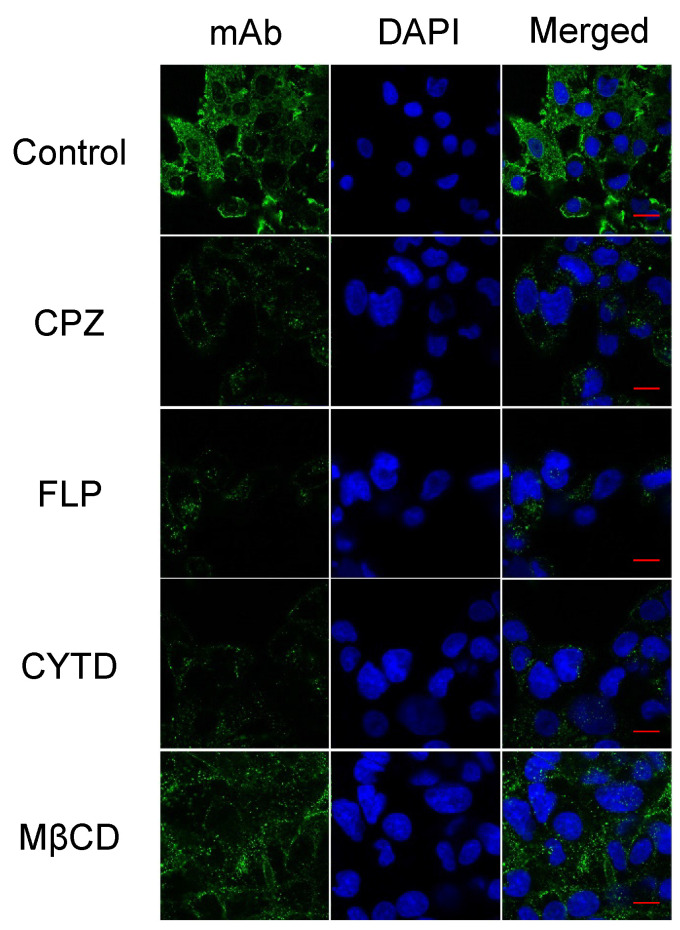
E. Corrected figure.

